# Personalized vs. population-based speech models for multi-dimensional mental health prediction

**DOI:** 10.3389/fdgth.2026.1690497

**Published:** 2026-06-09

**Authors:** Mashrura Tasnim, Jiayin He, Bo Cao, Eleni Stroulia

**Affiliations:** 1 Department of Computing Science, University of Alberta, Edmonton, AB, Canada; 2 Department of Psychiatry, Faculty of Medicine and Dentistry, University of Alberta, Edmonton, AB, Canada; 3 School of Public Health, University of Alberta, Edmonton, AB, Canada

**Keywords:** anxiety, convolutional neural network, depression, machine learning, mental health, personalized prediction, speech, stress

## Abstract

**Introduction:**

Mental disorders such as depression, anxiety, and stress are increasingly prevalent, particularly among young adults. Traditional assessment methods rely on self-reports and resource-intensive clinician interviews, limiting scalability and accessibility. Speech-based machine learning models offer a scalable and non-invasive alternative; however, population-level models often struggle to distinguish disorder-related signals from speaker-specific traits, reducing individual prediction accuracy.

**Methods:**

We propose a hybrid framework that combines population-level modelling with incremental individual-specific adaptation to improve personalized mental health prediction. The approach was evaluated using our longitudinal YouthDASS dataset, which contains over 1,000 speech samples collected up to two months from individuals aged 18-30 years, labelled with severity scores for depression, anxiety, and stress based on the DASS-21 scale. Multiple machine learning models were explored and assessed under population-only, individual-only, and hybrid modelling settings, among which a one-dimensional convolutional neural network (1D CNN) demonstrated the best performance.

**Results:**

The hybrid approach outperformed population-level models across all three mental health conditions, achieving lower individual-level root mean square error (RMSE) values of 6.95 for depression, 7.15 for anxiety, and 4.95 for stress on the DASS-21 scale. In comparison, individual-only models demonstrated mixed performance across disorders.

**Discussion:**

These findings suggest that integrating population-level knowledge with individual-specific adaptation provides a stronger balance between generalization and personalization than either approach alone. The proposed framework supports the development of scalable, personalized speech-based mental health monitoring systems and highlights the potential of adaptive machine learning methods for longitudinal mental health assessment.

## 1 Introduction

Mental disorders represent a growing global health crisis, particularly among adolescents and young adults. According to recent epidemiological reports, individuals aged 15–24 experience the highest prevalence of mental illness and substance use disorders, with approximately one in four affected [1, 2]. Depression, anxiety, and stress (DAS) are especially common in this age group, contributing to reduced academic performance, impaired social functioning, and elevated risk of self-harm. Traditional clinical assessments rely heavily on self-report questionnaires and clinician-administered interviews, which are resource-intensive and often fail to provide timely support. In this context, speech-based non-invasive monitoring systems offer a promising alternative: they are low-cost, scalable, and capable of enabling early detection and continuous support without placing additional burden on patients or clinicians.

Speech has long been studied as a non-invasive biomarker of mental health. Cummins et al. [3] provided an early survey highlighting how prosodic, spectral, and glottal features capture depression severity, though most work at that time emphasized binary detection rather than continuous symptom estimation. More recently, [4] argued that speech-based biomarkers show strong promise for scalable assessment, but challenges remain in generalization, fairness, and clinical validity. Low et al. [5] reached similar conclusions in a systematic review, calling for greater attention to regression-based approaches that track the severity of symptoms over time rather than categorical classification.

Several recent studies have moved toward the prediction of depression severity scores. Costantino [6] used support vector regression (SVR) and random forest regressors on the DAIC-WoZ speech recordings labelled with PHQ-8 scores, reporting that data scarcity and imbalance limit performance. Milintsevich et al. [7] applied a hierarchical regression framework combining linear regression and multi-target learning to predict multiple PHQ-9 symptom severities from transcripts, showing that modelling symptom dimensions jointly improved performance. Dai et al. [8] investigated elastic net regression and random forest regression for feature selection and severity prediction, finding that optimized feature subsets reduced RMSE compared to the full set of features. These works demonstrate the feasibility of regression models but highlight the need for improved feature engineering and modelling strategies.

An important limitation of population-level models is their difficulty in disentangling depression markers from stable speaker characteristics. Dumpala et al. [9] showed that acoustic features linked to depression severity overlap significantly with those used for speaker recognition, complicating generalization across individuals. To address such challenges, personalization strategies have gained attention. Gerczuk et al. [10] proposed zero-shot personalization using pretrained speech foundation models, demonstrating improved fairness at the individual level but requiring large-scale pretrained backbones. Kathan et al. [11] employed personalized recurrent neural networks (RNNs) on mobile sensing and ecological momentary assessment data, showing that subject-specific tuning improved predictive accuracy over population-level baselines. These findings support the development of hybrid approaches that combine population-level generalization with individual-level adaptation.

Although our work focuses on speech, related studies in other modalities reinforce the value of personalized regression. Weintraub et al. [12] used linear mixed-effects regression models on adolescent speech data, finding that linguistic patterns predicted depressive symptoms longitudinally and within-subject adaptation improved accuracy. Sadeghi et al. [13] explored transformer-based large language models combined with facial expression analysis for multimodal depression detection, concluding that multimodality and personalization are crucial for robust symptom estimation. Together, these studies suggest that personalized regression models, whether speech-only or multimodal, can yield more clinically relevant assessments than categorical classifiers. Comorbidity of depression, anxiety, and stress is highly prevalent, particularly in adolescents and young adults. Epidemiological studies indicate that these disorders frequently co-occur, often sharing overlapping symptomatology and risk factors [14, 15]. For example, individuals experiencing depressive symptoms are significantly more likely to report heightened anxiety and stress, and vice versa, complicating both diagnosis and treatment. From a modeling perspective, this comorbidity suggests that predictive systems focusing on one disorder in isolation may overlook critical interdependencies. By jointly modeling DAS severity, speech-based systems can more effectively capture the shared and distinct acoustic markers of these conditions. This not only improves predictive accuracy but also provides a more holistic representation of mental health, aligning with clinical realities where comorbid presentations are the norm rather than the exception.

In this study, we extend this line of research by developing personalized regression models for predicting depression, anxiety, and stress (DAS) severity from speech. Our hybrid approach integrates population-level models with individual-specific adaptation, aiming to balance generality with individual relevance. Our framework demonstrates how personalization can enhance predictive accuracy while remaining computationally efficient. In doing so, it addresses key limitations of earlier studies, particularly the challenges of generalization across speakers and the limited capacity of population-only models to capture individual variability, indicating the severity of depression, anxiety, and stress. Although thresholds exist for these disorders, we focus on predicting continuous symptom severity rather than binary classification, as this preserves the granularity of symptom information, allows monitoring of subclinical changes over time, and supports personalized intervention strategies—while still permitting downstream classification and evaluation with traditional metrics if needed.

## 2 Materials and methods

This study compares three approaches for predicting depression, anxiety, and stress scores from speech: the **Population Model**, the **Individual-Specific Model**, and the **Hybrid Model** to examine the **cross-subject effect**, the **within-subject effect** and the **mixed effect**, respectively. The rationale for this design is to examine the trade-off between generality across participants and personalization for individual users, a critical issue in building reliable mental health assessment systems [3, 16].

### 2.1 The YouthDASS dataset

YouthDASS is a longitudinal speech corpus containing over 1,000 speech samples in English and Spanish, collected from May 2022 to March 2023. The COVID-19 pandemic increased the prevalence of mental health disorders, and studies show that during the pandemic, youth (15 to 39 years) were more vulnerable to depression and anxiety disorders [17]. Our dataset was collected during the post-pandemic period, recruiting participants between 18 and 30 years old. This dataset captures a significant positive correlation among the disorder scores, which supports the fact that these disorders tend to be comorbid.

The YouthDASS dataset consists of speech samples collected from 40 participants from Mexico and Canada [18]. 26 of the participants were native Spanish speakers (14 female, 9 male, and 3 identified as other gender), and 14 were English speakers (6 male, 8 female). We developed an Android application for data collection. The application prompted the participants to record samples every three days. On these days, participants provided two speech samples: one from guided reading and one from free-form speech. At the same time, they completed the DASS-21 [15] questionnaire. For guided reading, the participant read out the paragraph “Please call Stella” [19] in English or Spanish. For the free-form speech task, the participants were asked two questions, randomly selected from a list of questions, including describing a memorable event, a hobby, or a favourite person. The data collection continued for two months, resulting in a corpus of 1,049 data points. 838 of the samples are in Spanish, and the rest are in English. Each audio sample ranges from 23 to 67 s in duration. We obtained 1 to 54 speech samples per participant, 26 on average (Table 1).

**Table 1:** Summary of the YouthDASS dataset demographics and data collection protocol.

Characteristic	Value/Description
Number of participants	40
Location	Mexico, Canada
Spanish speakers	26 (14 female, 9 male, 3 other)
English speakers	14 (8 female, 6 male)
Total speech samples	1,049
Spanish samples	838
English samples	211
Data collection duration	2 months
Sampling frequency	Every 3 days
Samples per session	2 (guided reading and freeform speech)
Speech duration	23–67 s
Samples per participant	1–54 (avg. 26)
Disorder scale	DASS-21

Every recording session is labelled with the DASS-21 [15] score, completed on the same day. The DASS-21 scale consists of 21 statements, with 7 questions associated with each of the scales of Depression, Anxiety, and Stress. The participants rated each statement from 0 to 3, indicating how much the statement applied to them. The three scales of DASS-21 provide scores on individual depression, anxiety, and stress in the range of 0 to 21; these scores are then multiplied by 2 for consistency with the more detailed DASS-42 scale [20]. The thresholds for being considered healthy on the depression, anxiety, and stress scales are scores below 9, 7, and 15 (out of 42), respectively. Based on these thresholds, 77%, 72%, and 88% of our samples fall within the normal range for depression, anxiety, and stress, respectively.

### 2.2 The study design

This study aims to investigate how the source of the training samples, namely the individual themselves or the general population in which they belong, influences the performance of machine learning models for predicting the target individual’s depression, anxiety, and stress from speech. Specifically, we seek to answer the following research questions:

1. How accurately can a machine learning model predict mental health outcomes when trained on speech samples from a general population, excluding the target participant?
2. What level of predictive accuracy can be achieved using speech samples exclusively from the target participant?
3. Can a hybrid approach, training a model on population data and then fine-tuning it using individual-specific samples, improve predictive performance compared to using either data source alone?

By comparing these three approaches, this study aims to provide insights into the relative effectiveness of these strategies and identify which approach performs best for building personalized yet generalizable models for speech-based mental health prediction.

Given our research questions, it is evident that accurate modelling depends on sufficient and balanced data from each participant. In order to determine the YouthDASS subset appropriate for our study, we examined whether each participant provided a sufficient number of samples for meaningful machine learning analysis. Because our model optimization was conducted using flat cross-validation with 3 splits, we required that each participant’s dataset allow for a balanced distribution of samples across folds. Specifically, participants were included only if their dataset contained at least six samples, with a minimum of three sessions corresponding to DASS-21 scores above the predefined threshold and a minimum of three sessions below the threshold. This criterion ensured that, within each cross-validation split, there was at least one sample in each category (above and below the threshold). Participants who did not meet these criteria were excluded from further analysis.

This results in 19, 15 and 17 participants for depression, anxiety, and stress, respectively. Among these, the ratio of English to Spanish speakers was 6:13 for depression, 6:9 for anxiety, and 8:9 for stress. Table 2 summarizes the descriptive statistics of DASS-21 scores in our final dataset.

**Table 2:** Descriptive statistics of the DASS-21 scores in each language after applying exclusion criteria.

Language	Metric	Depression	Anxiety	Stress
English	Mean ± Std. Dev.	8.14 ± 6.63	6.86 ± 4.68	10.76 ± 6.46
	Range	[0, 28]	[0, 24]	[0, 30]
	#Participants	6	6	8
	#Sessions	112	100	125
	#Sessions above minimum level threshold	43	41	36
	#Samples	221	194	243
Spanish	Mean ± Std. Dev.	8.71 ± 8.30	6.67 ± 6.83	13.40 ± 9.16
	Range	[0, 42]	[0, 42]	[0, 42]
	#Participants	13	9	9
	#Sessions	229	153	150
	#Sessions above minimum level threshold	84	61	63
	#Samples	457	307	302
Overall	Mean ± Std. Dev.	8.52 ± 7.80	6.74 ± 6.08	12.22 ± 8.17
	Range	[0, 42]	[0, 42]	[0, 42]
	#Participants	19	15	17
	#Sessions	341	253	275
	#Sessions above minimum level threshold	127	102	99
	#Samples	678	501	545

We acknowledge that these inclusion criteria do not guarantee a sufficiently large number of samples per participant to effectively train robust machine learning models. Moreover, the overall number of participants in each disorder group (15–19) is limited and therefore does not allow us to draw definitive conclusions about the effectiveness of the proposed personalization approach. In future work, we aim to collect a richer and more balanced dataset with a larger number of participants and samples per individual to enable more reliable training and evaluation of personalized models.

A key methodological decision in this study is how to partition data for training and testing, as this directly affects whether models capture general patterns across participants or individual-specific traits. To investigate this, we evaluate three modelling strategies:

1. **Population Model**: The population model evaluates the cross-subject effect by capturing patterns shared across participants. We implement it using leave-one-subject-out (LOSO) cross-validation: in each iteration, all samples from one participant are held out for testing, while the model is trained on the remaining participants’ data. This approach assesses how well the model generalizes across individuals and ensures it does not simply memorize speaker-specific traits.
2. **Individual-Specific Model**: The individual-specific model focuses on the within-subject effect, learning patterns unique to a single participant. Three-fold non-overlapping cross-validation is applied using only the target participant’s speech samples, preserving the ratio of disorder scores above and below threshold. This design captures individualized vocal markers of mental state, which are crucial for personalized monitoring, though it has limited generalizability to other participants.
3. **Hybrid Model**: The hybrid model combines population-level generalizations with individual-specific adaptation to investigate the mixed effect. It is implemented by fine-tuning the population model on two of the three folds from the left-out participant and testing on the remaining fold. This approach leverages general patterns learned across participants while adapting to unique vocal characteristics, enhancing predictive performance when individual-level data are limited.

It is important to note here that the longitudinal repeated-measures nature of the dataset introduces potential temporal autocorrelation within participants, where observations collected closer in time may be more similar than those further apart. However, depression, anxiety, and stress are episodic in nature and do not necessarily follow a monotonic progression over time; as such, symptom severity can fluctuate, and observations can be reasonably treated as conditionally independent for this analysis. Future work will incorporate time-aware validation strategies and temporal modelling techniques to better account for these dependencies.

### 2.3 The data preprocessing pipeline

The speech samples are processed through a four-stage pipeline to produce the feature vectors to be used as input to the machine-learning model construction.

#### 2.3.1 Speech preprocessing

To suppress background noise and variability arising from microphone settings in participants’ personal mobile devices, the *Noisereduce* (https://github.com/timsainb/noisereduce) algorithm was applied to the speech samples of the YouthDASS dataset. In addition, all recordings were amplitude-normalized to a Root Mean Square (RMS) level of −20 dBFS (decibels relative to full scale) to reduce variability caused by differences in speaking volume. This preprocessing ensures consistent signal quality across sessions and participants, improves feature comparability, and facilitates the identification of meaningful acoustic markers rather than recording artifacts [21].

#### 2.3.2 Audio feature extraction

Extracting robust and informative features from speech is central to predicting mental health states. Our pipeline includes both interpretable, theory-driven conventional acoustic features as well as deep learning embeddings in our experiment:

1. **eGeMAPS**: The extended Geneva Minimalistic Acoustic Parameter Set (eGeMAPS) consists of 88 features computed from low-level descriptors (LLDs) of the speech signal. The LLDs include pitch, jitter, shimmer, loudness, harmonics-to-noise ratio (HNR), spectral slope, alpha ratio, Hammarberg index, the frequencies and relative levels of formants 1–3, formant bandwidths, harmonic ratios (H1–H2, H1–A3), spectral energy proportions (0–500 Hz and 0–1000 Hz), MFCC 1–4, linear pitch, and spectral flux. Statistical functionals such as mean, standard deviation, percentiles, and slope are applied to the LLDs to compute the final features. The eGeMAPS set is minimalistic but designed to capture relevant paralinguistic and affective information from speech [21]. Using eGeMAPS allows us to extract interpretable acoustic markers that can be linked to mental health constructs, providing insights into which vocal characteristics correlate with depression, anxiety, or stress.
2. **VGG-19 deep spectrum features**: Deep spectrum features are extracted using the pre-trained VGG-19 convolutional neural network [22] via the DeepSpectrum toolkit [23]. Speech signals are segmented using Hamming windows of 16 ms with 8 ms shift, and the power spectral density is computed on the dB scale. Spectrograms of 387 × 387 pixels are generated using the *viridis* colormap (https://matplotlib.org/stable/gallery/color/colormap_reference.html) and resized to 224 × 224 pixels to match the input size of VGG-19. The spectrograms are forwarded through the network, and activations from the second fully connected layer are extracted as 4,096-dimensional feature vectors for each sample. These deep features encode non-linear, high-level patterns in speech that may reflect subtle markers of affective state not captured by hand-engineered features.

The use of both eGeMAPS and VGG-19 features ensures coverage of both interpretable acoustic cues and complex abstract representations, enhancing predictive performance.

#### 2.3.3 Cross-validation fold generation

Before training machine learning models, both feature sets are standardized, and the most relevant features are selected to ensure consistent scales and manageable dimensionality for training the machine learning models. We splitted the extracted features into training and test sets prior to performing these step as described in Section 2.2, to avoid any potential data leakage.

#### 2.3.4 Feature normalization

Z-score standardization (Equation 1) was applied to center and scale each feature of the eGeMAPS feature set:

$$z_{ij}=\frac{x_{ij}-\mu_{j}}{\sigma_{j}}$$ (1)

where $x_{ij}$ denotes the value of feature j for sample i, and $\mu_{j}$ and $\sigma_{j}$ represent the mean and standard deviation of feature j, respectively, computed from the training set. This transformation ensures that all features are on a comparable scale, preventing features with larger magnitudes from dominating the learning process. The statistics computed from the training set are consistently applied to the test set to avoid data leakage.

For VGG-19 features, some dimensions contain only zero values, which can lead to numerical instability in z-score standardization. Therefore, min–max normalization (Equation 2) was applied:

$$x_{ij}^{\prime }=\frac{x_{ij}-x_{j}^{\min}}{x_{j}^{\max}-x_{j}^{\min}}$$ (2)

where $x_{j}^{\min}$ and $x_{j}^{\max}$ denote the minimum and maximum values of feature j in the training data, respectively. This transformation scales each feature to the range [0,1], preserving the relative distribution of values while ensuring numerical stability for CNN-based inputs.

#### 2.3.5 Feature selection

After standardization, a subset of features was selected from the training data set using the Minimum Redundancy Maximum Relevance (mRMR) [24] criterion, which identifies features that are highly relevant to depression, anxiety, and stress scores while minimizing redundancy among them. Based on this criterion, we retained 50 features from eGeMAPS and 512 features from VGG-19. This feature selection step reduces computational cost, mitigates overfitting, and enhances model interpretability, while ensuring that the selected features capture the most informative acoustic cues for predicting depression, anxiety, and stress.

### 2.4 Machine learning models

The choice of model depends on the characteristics of the data and the objectives of the prediction task. In this study, this implies the requirement for architectures that effectively capture speech patterns indicative of mental health conditions. Specifically, we selected Random Forest and Convolutional Neural Network (CNN) models, as their architectures support incremental training, which enables the formulation of our proposed hybrid model.

#### 2.4.1 Random forest

We employ a random forest regressor to predict depression, anxiety and stress scores from the selected speech features from eGeMAPS feature set. Random forests are ensembles of decision trees that reduce variance through averaging, making them robust to overfitting, heterogeneous feature types, and non-linear relationships. We first use the parameter settings in the Scikit-learn implementation as our baseline model, establishing a performance benchmark without extensive tuning and ensuring reproducibility.

To improve upon baseline performance, we perform hyperparameter tuning to optimize model complexity, control overfitting, and maximize predictive accuracy as listed in Table 3. By systematically tuning these hyperparameters with the training data, we aim to balance bias and variance, enhance predictive performance, and ensure the random forest model effectively captures speech features associated with depression, anxiety, and stress.

**Table 3:** Hyperparameters of the random forest and CNN models.

Hyperparameter	Value(s)
Random forest	
Number of estimators (n_estimators)	100, 200, 300
Maximum depth (max_depth)	5, 10, 15
Min. samples to split (min_samples_split)	5, 10
Min. samples per leaf (min_samples_leaf)	10, 20, 40
Hybrid extension	+100 trees trained on individual data
CNN architecture	
Input dimension	512 feature vector
Conv1	512 filters, kernel = 5, stride = 1, ReLU
Conv2	256 filters, kernel = 5, stride = 1, ReLU
Max pooling	Pool size = 8
Dropout	0.2 (after conv block), 0.3 (dense layer)
Dense layer	256 units, ReLU
Output layer	1 neuron (regression output)
Training settings	
Optimizer	Adam
Initial learning rate	$1\times 10^{-6}$
LR schedule	Exponential decay (steps = 500, rate = 0.96)
Batch size	8
Epochs	Up to 300
Hybrid fine-tuning	Dense layer retrained for 20 epochs
Fine-tuning protocol	3-fold cross-validation per participant

For the hybrid model, we extend the population-trained random forest by adding 100 additional trees trained on two folds of the held-out participant’s data, with predictions made on the third fold, repeating three times. This allows the model to retain population-level knowledge while adapting to individual-specific vocal patterns, improving personalization without sacrificing generality.

#### 2.4.2 One-dimensional convolutional neural network (1D-CNN)

To model interactions within the selected VGG-19 speech features, we implement a one-dimensional convolutional neural network (1D-CNN). The network takes the 512-dimensional feature vector, obtained after mRMR selection, as input and applies a series of convolutional, pooling, and fully connected layers to predict DAS scores. The architecture of the model and the training parameters are summarized in Table 3. These settings are inspired by our previous experimental findings [18], chosen to ensure stable training, prevent overfitting, and enable the network to learn informative patterns from high-dimensional speech representations.

It is important to note that the selected features do not possess an inherent temporal or spatial ordering, as mRMR prioritizes feature relevance and redundancy rather than structural continuity. Consequently, the use of a 1D-CNN in this context is not intended to capture sequential or temporal dependencies. Instead, the convolutional layers act as learnable local feature combiners that can model interactions among neighbouring features in the embedding space. This design facilitates parameter sharing and hierarchical feature abstraction, offering a more compact alternative to fully connected architectures. We acknowledge that this represents a modelling approximation, and alternative architectures such as multilayer perceptrons (MLPs) could also be considered for this task.

**Figure 1:**
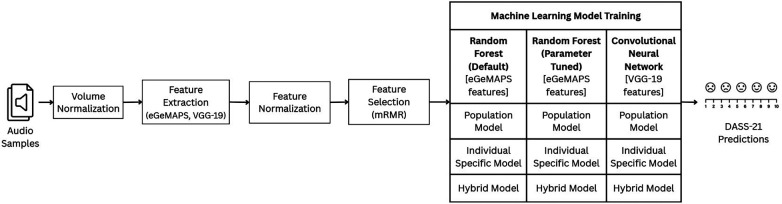
Workflow of the proposed framework for predicting depression, anxiety, and stress (DAS) severity from speech.

For the hybrid model, we leverage population-level training followed by individual-specific fine-tuning. First, the convolutional layers are trained on the data from all participants except one to capture generalizable patterns associated with DAS scores. In the fine-tuning phase, the convolutional layers are frozen, and only the fully connected dense layer is retrained using two of the three non-overlapping folds of the held-out participant’s data, with predictions made on the third fold. Fine-tuning is performed for 20 epochs, and the process is repeated three times to obtain predictions across all folds of the held-out participant. This approach enables the model to retain population-level knowledge while adapting to individual-specific variations in speech, thereby enhancing personalized prediction without compromising generalization. The overall workflow of our proposed system is illustrated in Figure 1.

All experiments were performed on a workstation with a 13th Gen Intel Core i9-13900F processor (2.00 GHz), 32 GB of system memory, and an NVIDIA GeForce RTX 4060 GPU.

It is important to note here that this selection is not intended to provide an exhaustive benchmark of all possible machine learning models. Rather, our goal is to compare personalization strategies across two representative model families: a classical machine learning approach (Random Forest) and a neural network-based approach (CNN). This design allows us to isolate and evaluate the impact of personalization within and across these distinct modelling paradigms.

### 2.5 Performance metrics

To evaluate the predictive performance of our models, we employ two widely used regression metrics: Root Mean Square Error (RMSE) and the coefficient of determination ($R^{2}$). Both metrics provide complementary insights into model performance and are appropriate for our task, where the objective is to predict continuous mental health scores (depression, anxiety, and stress) from speech features.

RMSE is suitable for our task because it indicates how closely the predicted scores align with the actual mental health scores. It penalizes larger errors more heavily, which is especially important in mental health assessments where large deviations may correspond to misclassification of individuals with significant conditions. Moreover, RMSE is expressed in the same units as the target variable, making it directly interpretable in the context of the original scores. Formally, RMSE is defined as:

$$\mathrm{RMSE}=\sqrt{\frac{1}{n}\sum_{i=1}^{n}{\left(y_{i}-{\hat{y}}_{i}\right)}^{2}}$$ (3)

where $y_{i}$ represents the observed values, ${\hat{y}}_{i}$ represents the predicted values, and n is the total number of samples (Equation 3).

The $R^{2}$ score quantifies how much of the variability in depression, anxiety, and stress scores can be explained by acoustic features, providing insight into the explanatory power of the models. An $R^{2}$ value of 1 indicates perfect predictions, while a value of 0 indicates the model performs no better than a naive mean predictor. Negative values suggest the model performs worse than this baseline. Formally, $R^{2}$ is defined as:

$$R^{2}=1-\frac{\sum_{i=1}^{n}{\left(y_{i}-{\hat{y}}_{i}\right)}^{2}}{\sum_{i=1}^{n}{\left(y_{i}-\bar{y}\right)}^{2}}$$ (4)

where $\bar{y}$ is the mean of the observed values (Equation 4).

## 3 Results

**Figure 2:**
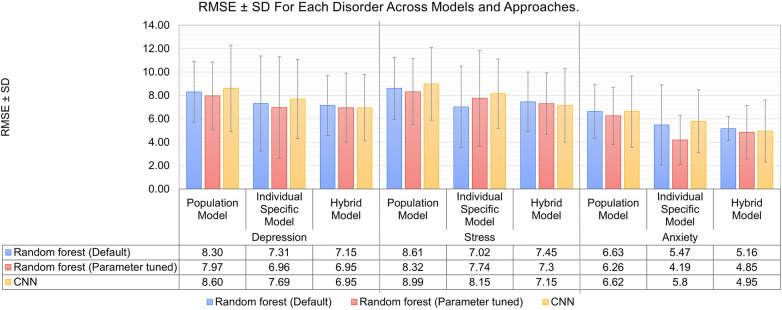
RMSE ± SD for each disorder across models and approaches. Hybrid models generally show improved performance.

Figure 2 summarizes the results across population-level, individual-specific, and hybrid models for default random forest, hyperparameter-tuned random forest, and CNN.

### 3.1 Do personalized models perform better than population models in predicting depression, anxiety and stress?

Across all three disorders, the hybrid models generally achieve the lowest RMSE and highest $R^{2}$, highlighting the benefit of combining population-level and individual-specific information. For example, for depression, the hybrid CNN achieves an RMSE of 6.95 ± 2.91, compared to 8.56 ± 3.81 for the population model and 7.69 ± 3.47 for the individual-specific model. Similar trends are observed for stress and anxiety, with hybrid models consistently showing reduced RMSE (Figure 2) and improved $R^{2}$.

Importantly, Table 4 shows that all population-level models yield negative $R^{2}$ values across disorders (ranging from −0.14 to −0.43), indicating that they perform worse than a naïve mean predictor. This result reflects a key limitation of population-based modelling in this setting. The dataset is highly imbalanced, with over 70% of samples corresponding to the healthy range of DAS scores. As a result, population models tend to predict values close to the overall mean, leading to systematic underestimation of higher symptom severity and poor overall fit.

**Table 4:** Coefficients of determination ($R^{2}$) for each disorder across models and approaches.

Disorder	Model	$R^{2}$		
		Population	Individual-specific	Hybrid
Depression	RF (Default)	−0.21	0.05	0.09
	RF (Tuned)	−0.14	0.09	−0.12
	CNN	−0.36	−0.07	**0.12**
Stress	RF (Default)	−0.23	**0.19**	0.13
	RF (Tuned)	−0.15	0.13	0.16
	CNN	−0.36	−0.06	**0.19**
Anxiety	RF (Default)	−0.41	0.21	0.19
	RF (Tuned)	−0.24	0.19	**0.26**
	CNN	−0.43	−0.07	0.18

Hybrid CNN models generally show a positive correlation with higher values (Except for Anxiety). Highest $R^{2}$ values are boldfaced.

In contrast, individual-specific models achieve positive $R^{2}$ values in most cases (up to 0.21 for anxiety), suggesting that they are better able to capture intra-individual variability and subtle acoustic changes associated with symptom fluctuations. Despite being trained on limited data, these models benefit from reduced heterogeneity within each participant.

Hybrid models achieve the best overall performance in most cases, with the highest $R^{2}$ values observed for tuned RF (0.26 for anxiety) and CNN (0.19 for stress) (Table 4). However, some exceptions remain. For instance, the tuned RF hybrid model underperforms the individual-specific variant for depression, and the CNN hybrid model shows comparatively lower gains for anxiety. These results suggest that while hybridization is generally effective, its benefits may depend on the model architecture and disorder-specific characteristics.

To quantify whether the observed performance improvements of the hybrid models were statistically significant, we conducted paired tests comparing hybrid models against population and individual-specific models. The choice of statistical test depends on the normality of the differences: we used the Shapiro–Wilk test to assess normality, followed by paired t-tests for normally distributed differences or Wilcoxon signed-rank tests otherwise.

Because multiple pairwise comparisons were performed, we applied a **Bonferroni correction** to control the family-wise error rate. Specifically, the significance level was adjusted from α=0.05 to α=0.0167 by dividing by the number of comparisons (n=3). Statistical significance was therefore determined based on this corrected threshold.

We excluded the default random forest from these tests because the tuned version consistently outperforms it, making comparisons with the tuned RF and CNN more meaningful. These analyses enable us to rigorously determine whether hybridization provides a significant advantage beyond the traditional population model. The summary of the analysis is presented in Table 5.

**Table 5:** Significance testing of performance improvement by hybrid models compared to population and individual-specific models. Normality was assessed with the Shapiro–Wilk test; based on the outcome, either paired t-test or Wilcoxon signed-rank test was applied. A **Bonferroni correction** was performed to account for multiple comparisons, and adjusted significance thresholds (α=0.0167) were used. Significant improvements are shown in boldface.

Model	Disorder	Comparison	Normality (Shapiro–Wilk)	Test & Result	Interpretation
CNN	Depression	Hybrid vs. Population	Normal (p=0.067)	t=−5.65, p=0.0000	**Hybrid significantly better**
		Hybrid vs. Individual-Specific	Normal (p=0.443)	t=−2.81, p=0.0116	**Hybrid significantly better**
	Anxiety	Hybrid vs. Population	Normal (p=0.928)	t=−4.38, p=0.0006	**Hybrid significantly better**
		Hybrid vs.Individual-Specific	Normal (p=0.159)	t=−3.43, p=0.0040	**Hybrid significantly better**
	Stress	Hybrid vs. Population	Normal (p=0.281)	t=−4.56, p=0.0003	**Hybrid significantly better**
		Hybrid vs. Individual-Specific	Normal (p=0.600)	t=−2.51, p=0.0233	No significant difference
RF (Tuned)	Depression	Hybrid vs. Population	Non-normal (p=0.012)	W=3.0, p=0.0000	**Hybrid significantly better**
		Hybrid vs. Individual-Specific	Normal (p=0.582)	t=−0.03, p=0.9786	No significant difference
	Anxiety	Hybrid vs. Population	Normal (p=0.537)	t=−4.47, p=0.0005	**Hybrid significantly better**
		Hybrid vs. Individual-Specific	Non-normal (p=0.038)	W=36.0, p=0.1876	No significant difference
	Stress	Hybrid vs. Population	Non-normal (p=0.001)	W=3.0, p=0.0001	**Hybrid significantly better**
		Hybrid vs. Individual-Specific	Normal (p=0.888)	t=−0.66, p=0.5170	No significant difference

Overall, the hybrid approach shows statistically significant improvements over population-level models across all disorders and model types (all p≤0.0167 after Bonferroni correction). When compared to individual-specific models, hybrid CNNs achieve significant gains for depression (p=0.0116) and anxiety (p=0.0040). In contrast, parameter-tuned hybrid random forest models do not show significant improvement over individual-specific models for depression (p=0.9786), anxiety (p=0.1876) or stress (p=0.5170).

Across all three disorders, the hybrid model consistently improves upon the population model, reducing RMSE and increasing $R^{2}$. However, the improvement over the individual-specific model is smaller and, in some cases, not statistically significant, particularly when sufficient individual-level data is available. This pattern suggests that hybridization primarily benefits scenarios where the population model alone is limited, while its added value diminishes as more individual-specific samples are included.

### 3.2 How do different model personalization approaches compare?

To assess whether the observed differences in individual-level RMSE between CNN and random forest models were statistically significant, we conducted paired statistical tests separately for each disorder and model type (Population, Individual, and Hybrid). Since the RMSE values are computed on the same participants, paired tests are appropriate. For each comparison, we first assessed the normality of the paired differences using the Shapiro–Wilk test. If the normality assumption was satisfied (p>0.05), a paired t-test was applied; otherwise, the non-parametric Wilcoxon signed-rank test was used. As multiple comparisons were performed within each disorder (three comparisons per disorder), a Bonferroni correction was applied to control the family-wise error rate. The significance level was adjusted from α=0.05 to α=0.0167. Statistical significance was assessed using this corrected threshold. The results of these tests are summarized in Table 6.

**Table 6:** Statistical comparison between CNN and RF models across different disorders and model types. The applied test depends on the normality of differences. Bonferroni-corrected significance level: α=0.0167 has been used for comparison. Statistically significant results are shown in bold.

Disorder	Model type	Test	Statistic	p-value	Interpretation
Depression	Population	Paired t-test	1.408	0.1761	No significant difference
	Individual	Paired t-test	1.261	0.2233	No significant difference
	Hybrid	Wilcoxon	75.500	0.4413	No significant difference
Stress	Population	Paired t-test	1.692	0.1100	No significant difference
	Individual	Paired t-test	0.537	0.5988	No significant difference
	Hybrid	Wilcoxon	44.500	0.1324	No significant difference
Anxiety	Population	Paired t-test	0.877	0.3953	No significant difference
	Individual	Wilcoxon	**14.000**	**0.0067**	**CNN significantly better**
	Hybrid	Wilcoxon	59.000	0.9780	No significant difference

The statistical analysis indicates that, after applying Bonferroni correction, CNN and random forest models performed comparably in terms of individual-level RMSE across most configurations. The only statistically significant difference was observed for anxiety prediction in the individual-specific setting, where CNNs significantly outperformed random forests. This finding suggests that CNNs may capture individual-level nuances in anxiety-related speech patterns more effectively than random forests, while for depression and stress, the two approaches achieved similar accuracy across all modelling strategies. Further investigation, including effect size analysis, would help quantify the magnitude and practical relevance of this difference.

For all three disorders, the $R^{2}$ values show a similar trend to RMSEs, with CNN and tuned random forest exhibiting comparable predictive accuracy, though hybrid CNNs tend to achieve higher $R^{2}$ for depression and stress, while tuned hybrid random forest marginally outperforms CNN for anxiety.

### 3.3 How effective are personalized models in predicting depression, anxiety and stress?

**Figure 3:**
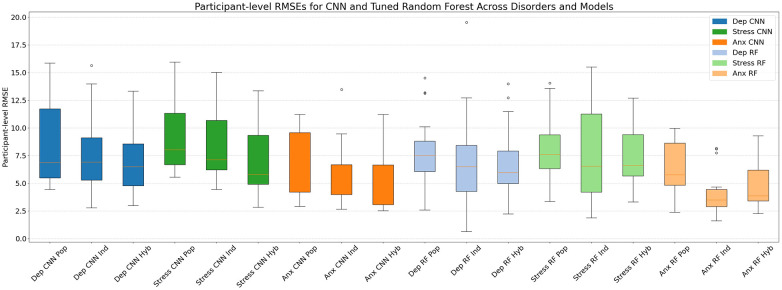
Individual-level RMSE distributions for CNN and tuned Random Forest across three disorders (Depression, Stress, Anxiety) and modelling approaches (Population, Individual, Hybrid).

Figure 3 illustrates the distribution of RMSEs at the individual level for CNN and tuned Random Forest models. Across all three disorders, the hybrid models generally achieve lower median RMSEs, confirming that combining population-level and individual-specific information improves predictive performance. For depression and stress, the hybrid CNN shows consistently lower RMSEs compared to the population and individual CNN models. Tuned Random Forest exhibits similar trends, though some participants show higher variability, particularly in individual models for stress.

The plot also highlights disorder-specific differences: anxiety predictions generally have lower RMSE ranges, reflecting the relative predictability of this condition. In most cases, the CNN model demonstrates slightly better performance than the tuned Random Forest in hybrid settings, which aligns with the observed $R^{2}$ trends indicating superior explanatory power of hybrid CNN models.

## 4 Discussion

Table 7 presents a comparison of our best-performing models with prior regression models for predicting the severity of depression, anxiety or stress. Different studies use various scales for measuring disorder severity, such as PHQ-8, PHQ-9, BDI-II, and DASS-21 and also different performance metrics to report performance of their regressors, including mean absolute error (MAE) and RMSE. To ensure fair comparison, we report normalized error, calculated as the ratio between RMSE or MAE and the full scale range. Normalized error ranges from 0 to 1, with values closer to 0 indicating better model performance. This normalization enables direct benchmarking of models’ outcomes presented in existing literature. However, due to differences in assessment scales, data modalities, participant populations, and languages, these comparisons should be considered illustrative rather than definitive, and caution is warranted when interpreting cross-study differences.

**Table 7:** Comparison of regression-based studies predicting disorder severity using normalized error (N-RMSE).

Study reference	Disorder	Sample size	Scale (Range)	Metric	Error	N-RMSE
Dai et al. [8]	Depression	189 participants	PHQ-9 (0–27)	RMSE	5.11	0.204
Costantino [6]	Depression	189 participants	PHQ-8 (0–24)	MAE	3.45	0.144
Kathan et al.[11] [Multimodal]	Depression	65 participants	PHQ-2 (0–6)	MAE	1.35	0.225
Gerczuk et al. [10]	**Depression**	143 participants (50,779 samples)	PHQ-9 (0–27)	MAE	0.98	**0.036**
Sadeghi et al. [13] [Multimodal]	Depression	275 participants	PHQ-8 (0–24)	MAE	4.20	0.175
Tasnim et al. [18]	Depression	40 participants (1,049 samples)	DASS-21 (0–42)	RMSE	7.09	0.169
Tasnim et al. [18]	Anxiety	40 participants (1,049 samples)	DASS-21 (0–42)	RMSE	7.69	0.183
Tasnim et al. [18]	Stress	40 participants (1,049 samples)	DASS-21 (0–42)	RMSE	8.40	0.200
Our hybrid CNN model	Depression	19 participants (678 samples)	DASS-21 (0–42)	RMSE	6.95	0.165
Our hybrid RF model	**Anxiety**	15 participants (501 samples)	DASS-21 (0–42)	RMSE	4.19	**0.100**
Our hybrid CNN model	**Stress**	17 participants (545 samples)	DASS-21 (0–42)	RMSE	7.02	**0.167**

Note that [11, 13] employed multimodal approaches. Lowest N-RMSE values are boldfaced for each disorder.

Our personalized models achieve competitive or superior performance compared to prior regression-based approaches. For depression, our best-performing hybrid CNN model achieved normalized RMSE of 0.167, outperforming population-level regression models such as those of [8, 11, 13, 18]. Some relevant works adapt multimodal approaches: [11] using mobile sensing and ecological momentary assessment data, and [13] combining large language models with facial expression analysis. Although these studies demonstrate strong performance, they rely on multi-modality and extensive sensing pipelines. In contrast, our speech-only framework demonstrates the feasibility of predicting depression, anxiety, and stress severity using a single easy-to-acquire modality. This highlights the computational efficiency and practical deployability of our method, though future work may benefit from incorporating multimodal signals to further improve accuracy and robustness. Recent zero-shot personalization approaches based on speech foundation models [10] report stronger performance than ours, largely due to the advantage of massive pretraining on diverse speech corpora and access to auxiliary metadata. However, such methods come at the cost of high computational requirements and reduced accessibility for lightweight clinical or mobile applications.

Speech-based regression models for predicting anxiety and stress remain relatively scarce in the literature, with most studies focusing primarily on depression or generalized mental health assessment. In this context, our current personalized modeling approach demonstrates a meaningful advancement. Compared to our previous non-personalized framework [18], the personalized models exhibit superior predictive performance for both anxiety and stress scores, highlighting the benefits of incorporating individualized vocal patterns in speech-based assessments.

A key strength of our framework lies in its personalized hybrid modeling strategy, which combines the generalization capacity of population-level models with individual-level adaptation. This balances robustness and sensitivity to individual variation, addressing the challenge noted by Dumpala et al. [9], who showed that depression markers often overlap with speaker identity traits. The personalized hybrid approach is particularly valuable for implementing ecological momentary interventions (EMIs), where speech-based assessments are delivered in real time during patients’ daily lives to provide just-in-time adaptive support [25, 26]. Unlike traditional clinical assessments that rely on retrospective self-reports during scheduled appointments, our framework can potentially enable continuous monitoring that captures the dynamic nature of mental health symptoms as they fluctuate throughout daily activities. This capability aligns with evidence that mobile health interventions can enhance therapeutic outcomes when personalized feedback is integrated with ongoing clinical care, offering benefits for both standalone and adjunctive treatment approaches [27].

Our system is also computationally efficient. While training the population-level model requires substantial data, time, and computational resources, the fine-tuning step is lightweight, updating only a small subset of model parameters. This makes fine-tuning considerably more efficient than retraining a full model or training individual-specific models from scratch. Although we did not perform formal benchmarking of training or inference times, the results suggest that fine-tuning can be performed efficiently, supporting potential real-time adaptation in practical settings. Comprehensive evaluation of computational performance is planned as part of future work.

## 5 Conclusion

This work demonstrates that personalized speech-based regression models can provide accurate and practical estimates of depression, anxiety, and stress severity. By leveraging a hybrid modeling strategy that integrates population-level generalization with individual-specific adaptation, our approach achieves competitive performance while maintaining computational efficiency and real-world deployability. The results further highlight the feasibility of using a single, non-invasive modality for continuous mental health monitoring, with potential applications in ecological momentary interventions and scalable digital health systems.

Nevertheless, several limitations should be considered. First, the sample size of participants was relatively small, which may limit the generalizability of our findings. Future studies with larger and more diverse cohorts are needed to confirm the robustness of the models across different populations.

Second, while personalization improved prediction accuracy, it requires sufficient individual-level data, which may not always be available in practical deployments. Future work could address this challenge through few-shot adaptation or transfer learning techniques.

Third, although linguistic features were not used, prior work suggests that prosodic and acoustic expressions of affect can vary across languages. In our current analysis, language was not explicitly modelled as a covariate in the learning process. As such, potential cross-linguistic differences in acoustic markers may still have influenced model performance. Future work will incorporate language-aware modelling and conduct more rigorous cross-linguistic validation using balanced datasets.

Finally, the dataset is characterized by an imbalanced distribution of symptom severity, with the majority of samples falling within the normal range of DASS-21 scores. While our regression-based approach aims to preserve symptom granularity, this imbalance may bias models toward predicting values near the healthy range and limit sensitivity to higher symptom severity. As a result, model performance in populations with greater prevalence or more severe symptom distributions may differ. Future work should evaluate these models on more clinically representative cohorts to better assess their utility in real-world mental health settings.

Looking ahead, an important direction for future research is the integration of multimodal signals, such as facial expressions, movement patterns, and physiological data. Incorporating complementary modalities may further enhance model robustness and resilience to noise, particularly in real-world deployment scenarios.

## Data Availability

The data analyzed in this study is subject to the following licenses/restrictions: The data used in this study was collected by our research group and is not publicly available. Requests to access these datasets should be directed to Mashrura Tasnim: mashrura@ualberta.ca, Eleni Stroulia: stroulia@ualberta.ca.
